# Analyzing hidden populations online: topic, emotion, and social network of HIV-related users in the largest Chinese online community

**DOI:** 10.1186/s12911-017-0579-1

**Published:** 2018-01-05

**Authors:** Chuchu Liu, Xin Lu

**Affiliations:** 1 College of Information System and Management, National University of Defense Technology, Changsha, 410073 China; 2 School of Business Administration, Southwestern University of Finance and Economics, Chengdu, 610074 China; 3 Department of Public Health Sciences, Karolinska Institutet, 17 177 Stockholm, Sweden

**Keywords:** Hidden population, Online community, HIV, Social network, Baidu Tieba, China

## Abstract

**Background:**

Traditional survey methods are limited in the study of hidden populations due to the hard to access properties, including lack of a sampling frame, sensitivity issue, reporting error, small sample size, etc. The rapid increase of online communities, of which members interact with others via the Internet, have generated large amounts of data, offering new opportunities for understanding hidden populations with unprecedented sample sizes and richness of information. In this study, we try to understand the multidimensional characteristics of a hidden population by analyzing the massive data generated in the online community.

**Methods:**

By elaborately designing crawlers, we retrieved a complete dataset from the “HIV bar,” the largest bar related to HIV on the Baidu Tieba platform, for all records from January 2005 to August 2016. Through natural language processing and social network analysis, we explored the psychology, behavior and demand of online HIV population and examined the network community structure.

**Results:**

In HIV communities, the average topic similarity among members is positively correlated to network efficiency (*r* = 0.70, *p* < 0.001), indicating that the closer the social distance between members of the community, the more similar their topics. The proportion of negative users in each community is around 60%, weakly correlated with community size (*r* = 0.25, *p* = 0.002). It is found that users suspecting initial HIV infection or first in contact with high-risk behaviors tend to seek help and advice on the social networking platform, rather than immediately going to a hospital for blood tests.

**Conclusions:**

Online communities have generated copious amounts of data offering new opportunities for understanding hidden populations with unprecedented sample sizes and richness of information. It is recommended that support through online services for HIV/AIDS consultation and diagnosis be improved to avoid privacy concerns and social discrimination in China.

**Electronic supplementary material:**

The online version of this article (10.1186/s12911-017-0579-1) contains supplementary material, which is available to authorized users.

## Background

A population is “hidden” when no sampling frame exists and public acknowledgment of membership in the population is potentially threatening [1–3]. As a representative of hidden populations, people who are infected with HIV/AIDS tend to suffer pressure and discrimination. However, due to social environmental pressure and other factors, there are many difficulties in conducting comprehensive and representative studies of the HIV population. To date, the study of this population has mainly focused on interviews and questionnaire surveys based on offline or online population sampling. In most cases, these traditional methods are inefficient, limited in sample size and representativeness, and challenged by privacy concerns and reporting error [4–8].

As a result of the development of Internet technology, people’s social lives have undergone tremendous changes from offline to online. People frequently publish, send, and share information in various virtual communities [9], thereby generating large amounts of data concerning online activity, which can be useful for the study of hidden populations. Through analyzing such data, it is possible to excavate the behavior patterns of hidden groups effectively, particularly as the number of online community users is unprecedentedly large, and it has been found that people are usually more honest and trusting when talking online [10–12]. It is expected that characteristics excavated from large-scale online community data may be more reliable, representative, and broad than those derived from offline data.

Hidden populations are gathered in all kinds of virtual communities, such that many scholars recruit or investigate hidden populations online [13, 14], especially recruiting respondents through links of online social networks [15]. In recent years, there have been many studies of hidden populations that have used snowball sampling and respondent-driven sampling (RDS) in online communities [16–18]. However, studies of hidden populations that have directly analyzed their online data in virtual communities are rare, and existing studies have mostly investigated social support for the targeted population. For example, Winefield examined the content and frequency of messages in an Internet support group to analyze the emotional support of women with breast cancer [19]. Im et al. used thematic analysis to explore the social support of patients with cancer in Internet cancer support groups (ICSGs) through an online forum [20]. Coursaris conducted content analysis of postings from a selected online HIV/AIDS forum to assess the types and proportions of social support exchanged among the HIV population [21]. Instead of discussing social support for hidden populations, in this study we try to understand the multidimensional characteristics of a hidden population by analyzing the massive data generated in the largest Chinese online community, Baidu Tieba. Specifically, we aim to extract features of the online users in the HIV group with regard to various aspects, including temporal patterns of online activity, social network structure, community structure and its connection to social distance and similarity of content, emotional tendency, etc. Most of these characteristics are typically difficult to study with traditional survey methods. Therefore, online data mining serves as an important supplement for the study of hidden populations, allowing researchers to investigate multidimensional characteristics of hard-to-access groups with unprecedented richness of information.

## Methods

### Data sources

As the world’s largest Chinese online community, Baidu Tieba has attracted a large number of social groups based on common interests [22]. Baidu Tieba is provided by Baidu, the dominant Chinese search engine company established on December 3, 2003. It functions by having users search or create a bar (Forum) by typing a keyword. If the bar has not yet been created, it is then created upon the search. “Bar” refers to a forum providing a place online where users can interact, covering topics related to games, films, popular stars, books, news, diseases, etc. Currently, Baidu Tieba has more than 20 million bars and the number of active users has reached 300 million [23].

**Fig. 1:**
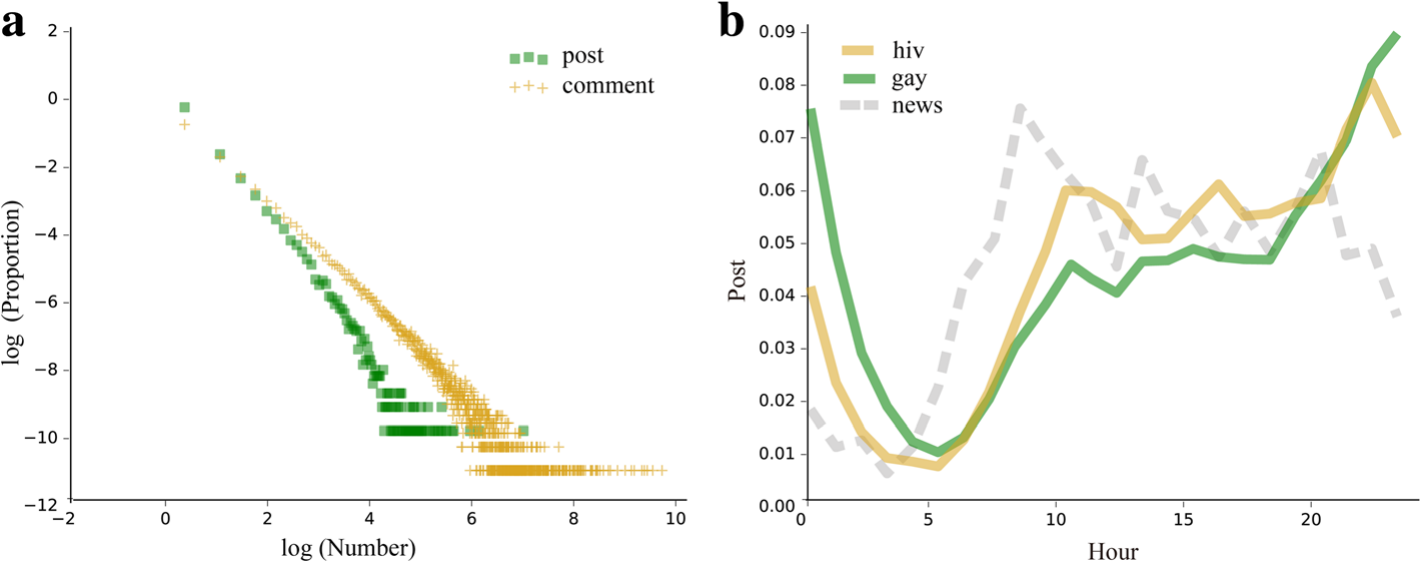
Basic statistics of online posting. **a** Distribution of the number of posts and comments. The horizontal axis represents the number of users’ posts or comments, and the vertical axis represents the frequency. **b** Comparison of posting time among different populations. The horizontal axis represents hours in the day, and the vertical axis represents the average proportion of posts generated in each hour

To collect activity data on the HIV population in the online community, we chose the largest bar related to HIV on Baidu Tieba, “HIV bar” (http://tieba.baidu.com/f?kw=hiv), and used Scrapy, a fast web-crawling framework, to extract the data we needed from the webpages. By elaborately designing the crawler, we were able to retrieve a complete dataset from the HIV bar for all records from January 2005 (the time when it was created) to August 2016. The dataset contains user information, content of posts, and the complete text of comments and replies. The collected data are saved in the local PostgreSQL database as three tables (Table 1), including a total of 72,328 user records, 76,865 posts, and 1,726,227 comments. There is considerable heterogeneity in the number of posts and comments generated by each user: while the majority (80%) of users wrote fewer than 4 posts and 15 comments, a small proportion of users actively generated a large number of posts and comments. The distribution of users’ comments and replies is shown in Fig. 1a.

**Table 1:** Data fields extracted from the HIV bar

HIV_User	HIV_Comment	HIV_Reply
User_id (user’s id)	Comment_id (comment id)	Reply_id (reply id)
Name (user’s name)	Post_id (post to which it belongs)	Comment_id (comment to which it belongs)
Sex (user’s sex)	Floor (the floor in its post)	Author_id (author’s id)
Level (user’s level in HIV bar)	Author_id (author’s id)	Reply_to_whom (user the author replies to)
URL (URL of user’s homepage)	Content (comment content)	Content (reply content)
	Time (comment time)	Time (reply time)
	Reply_num (number of replies)	
	Open_type (terminal type)

Based on the time of users’ posting in the HIV bar, we can analyze the temporal characteristics of the online HIV-related group and differences from other groups. As shown in Fig. 1b, compared with the news-related users and men who have sex with men (MSM)-related users (based on a large archive of data retrieved from MSM-related bars and news-related bars, including 270,229 users and 6,316,158 posts, respectively), the peak time of posting for HIV-related users is 22:00–23:00, and the lowest period is 3:00–5:00. It is worth mentioning that while the posts of ordinary users (news-related) decline from 20:00, those for the two representative hidden populations, MSM-related and HIV-related users, are on the rise. Initial inspection of the posts reveals that for ordinary users, their online topics are around news concerning politics, the economy, and social issues. For MSM-related users, their motivation for online posting is mostly for entertainment and to meet partners, and they are more active around midnight. For HIV-related users, their online topics are mostly related to consultation about HIV/AIDS, and as they are more concerned about their health status, they tend to go to sleep earlier than MSM-related people. More sophisticated analysis of the online content can be found in the Results section.

### Community mining

**Fig. 2:**
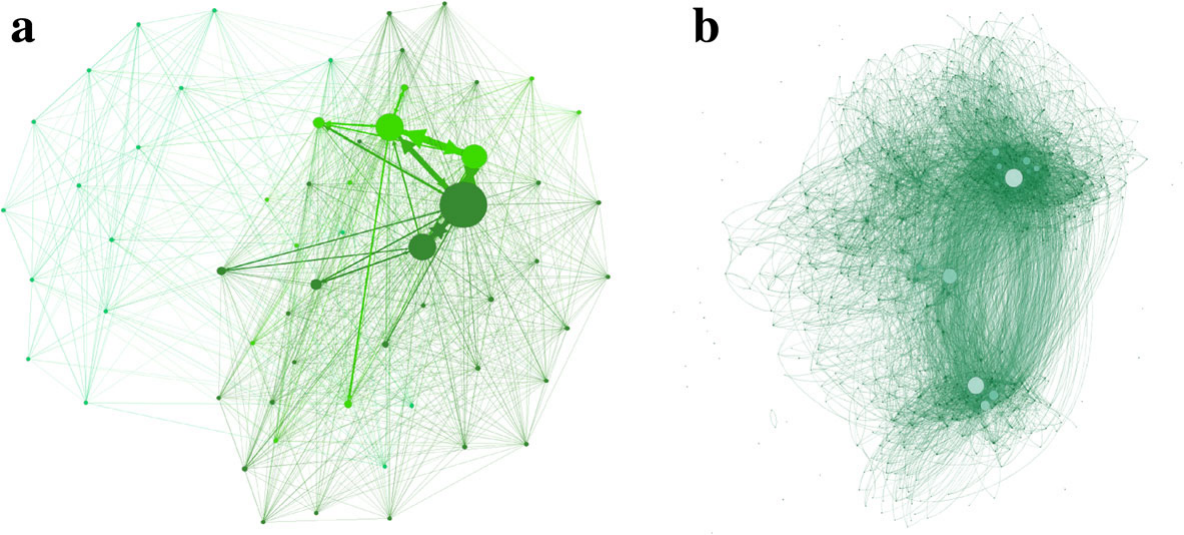
Interaction network of the HIV bar. **a** Community network. Nodes represent user communities and links represent interactions between users from the two connected communities; the size of the node is proportional to the number of users in each community. Communities with fewer than 50 users and links with fewer than 10 interactions between two communities are filtered out. **b** User network of a community. Nodes represent users and links represent interactions (replies or comments) among users; the size of the node is proportional to its degree

**Fig. 3:**
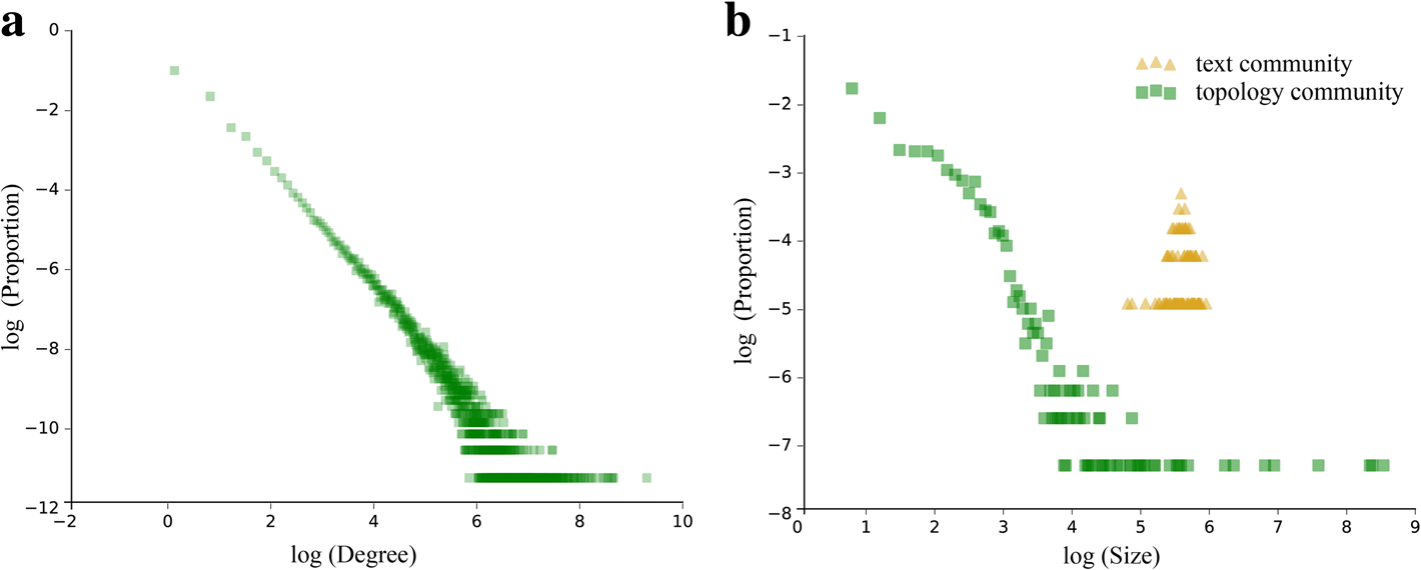
Distribution of network degree and community size**. a** Degree distribution of the interaction network. The horizontal axis represents the degree and the vertical axis represents the proportion of nodes. **b** Distribution of the size of communities. The horizontal axis represents the size of the community, and the vertical axis represents the proportion of communities

For many online activities, it has been shown that users tend to interact with others who are similar to themselves, forming distinct network communities, and stimulating studies on influence-based contagion or homophily-driven diffusion [24–27]. However, in-depth examination of the characteristics and dynamics of online community structure for hidden population was rarely found in literature [28, 29]. To fill in this gap of knowledge, in this study we explore possible communities in the HIV population who are active in the HIV bar, and analyze the characteristics and links of different communities to study the organizational model and behavioral characteristics of the HIV population. Using the response and comment relationships between users in the HIV bar as links and users as nodes, we construct an interaction network of the HIV population (hereinafter interaction network, see Fig. 2). As can be seen from Fig. 3a, the degree distribution of this network follows a power law, indicating large heterogeneity in the number of users with which each node interacts.

Using community detection in concert with topic modeling is a useful way to characterize communities for online population [30]. In this study, we implement community mining from two perspectives. First, we classify the users according to the content of their posts, and then discover user communities according to the topological structure of the interaction network. It is worth noting that the clustering based on text similarity only focuses on the content of users’ posts, regardless of links in the interaction network. After extracting all the content in the posts of active users (who have written more than three posts) in the HIV bar, we start the preprocessing of text, such as text cleaning and word segmentation, then use the Doc2Vec algorithm to construct the feature vectors of documents [31]. Finally, we implement text clustering with the unsupervised algorithm, K-means, to divide the users into groups with similar features. Since the K-means needs to determine the number of clusters manually, we use the sum of distances from all nodes to their cluster centers, as a criterion to select the best number of clusters:

$$ SSE=\sum \limits_{i=1}^k\sum \limits_{x\in {C}_i}\mid {\left|x-{u}_i|\right|}^2 $$

where *k* denotes the number of clusters, *u*_*i*_ denotes the cluster center in cluster *C*_*i*_*,* and ∣|x − *u*_*i*_|∣ denotes the distance between node *x* and the corresponding cluster center *u*_*i*_. *k* is then determined by minimizing *SSE*.

We also carry out community mining from the perspective of the network topology. Based on the structure of the users’ interaction network, we choose Infomap [32, 33], which is a highly efficient algorithm for detecting non-overlapping communities in directed weighted networks [34, 35], to detect communities in the interaction network. The sizes of the two groups of communities we found are shown in Fig. 3b. We can see that while the sizes of text similarity-based communities are all quite similar, the interaction network-based communities exhibit a wide range of sizes.

To explore the relationship between the text clusters and topological communities, we first use topic modeling algorithm to extract the topics of documents and measure the similarities of topics among users in the same cluster. Since the Latent Dirichlet Allocation (LDA) model [36, 37] requires the specification of the number of topics, the Hierarchical Dirichlet Process (HDP) model [38, 39], which is derived from LDA and can automatically determine the optimal number of topics, is used in the process of topic extraction in this study. Then for each cluster, we calculate the average topic similarity, which is the average of the similarities between all pairs of users in a cluster:

$$ S\ (C)=\frac{2}{n\ \left(n-1\right)}{\sum}_{i,j\in C}s\left(i,j\right) $$

where *n* denotes the number of users in a cluster *C*, and *s*(*i*, *j*) denotes the topic similarity between a user *i* and another user *j*. To measure the social distance of users in each community, we calculate the network efficiency [40], which is defined as:

$$ E\ (G)=\frac{2}{n\ \left(n-1\right)}\ {\sum}_{i<j\in G}\frac{1}{d\ \left(i,j\right)}. $$

where *d*(*i*, *j*) denotes the length of the shortest path between a node *i* and node *j*. In the figures of this paper, the average topic similarity is denoted by *S* and network efficiency is denoted by *E*.

### Text mining

To analyze popular terms (vocabulary) in HIV communities and the context in which these words are used, we use keyword discovery to extract the words the HIV population frequently posts online. It is worth mentioning that these HIV communities are those found by community mining based on the content of posts. In this study, we discover popular keywords according to their TF − IDF values. Based on the word segmentation results, we can calculate the TF − IDF value of each word, so the popular keywords can be selected after removing stop words. For the purpose of this study, we define the popular keywords as the top 100 meaningful words with the largest TF − IDF value in a community.

In addition, to analyze the topics the HIV population tends to discuss, and the purpose of the members’ online activity, topic detection is then carried out. Topics are discovered using the HDP model and we develop document clusters based on topic similarity. Thereby, we can conveniently identify the themes of clustered documents, i.e., the topics addressed by different users.

### Sentiment analysis

Sentiment analysis concerns analysis, processing, induction, and reasoning related to emotional subjective text aimed at discovering the attitude of the speakers on certain topics or their emotional state. By mining the text content of posts of users in the HIV bar, we can analyze the emotional state of this group. While both supervised learning and unsupervised learning can be used in this case [41–43], in this study, we mainly adopt the rule-based method to analyze the emotions of each user in the HIV group and the tendency in sentiment of different communities to uncover the emotional characteristics of the HIV population [44]. Sentiment words extraction is mainly based on two popular Chinese sentiment dictionaries, the Hownet lexicon and the National Taiwan University Sentiment Dictionary (NTUSD), both have approved ability of achieving high precision in the Chinese sentiment analysis [45, 46].

All posts by a user consist of a document. According to the text of each document, we extract the sentiment words, then calculate the sentiment score based on the frequency and intensity of sentiment words it contains. Positive words score from 1 to 5, negative words score from −1 to −5, and the absolute values represent sentiment intensities. If the sum of positive scores is greater than the sum of negative scores, the document is considered positive. Finally, each community is assigned a positive and negative score, representing the percentages of positive and negative users, respectively.

The precision and correctness of the dictionary-based sentiment analysis are further validated with a comparison to human judgments on a sample of 100 posts randomly selected from the data. As one can see from Table 1, Additional file 1: Table S2 and Table S3, the precision and recall rate are above 85% and 89%, respectively.

## Results

### Community mining based on text similarity

**Fig. 4:**
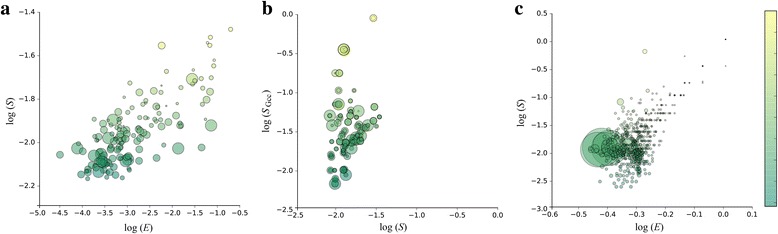
Correlation between content and network structure**. a** Correlation between network efficiency and average topic similarity in text-based communities; **b** the relationship between topic similarities in communities and topic similarities in the maximum connected component (denoted by S_Gcc in text-based communities); **c** correlation between network efficiency and the average topic similarity in topology-based communities. The size of scatter represents the size of community and the color corresponds to the level of topic similarity (green, low; yellow, high)

Using text clustering we find 150 clusters, each of which corresponds to a network (community) formed by interaction between users. We find a positive correlation between the average topic similarity of each cluster and the network efficiency of the cluster’s corresponding community, as shown in Fig. 4a. The correlation coefficient is *r* = 0.70 (*p* < 0. 001, nonlogarithmic, the same below), indicating that the higher the network efficiency of the text-based community (cluster), the greater the average topic similarity. That is, the closer the association within the community, the more similar the topics the community members discuss. Moreover, the average topic similarity of each text-based community also shows a positive correlation with the size of the largest weakly connected component [47], the maximal sub-graph in which for every pair of vertices there is an undirected path, and the correlation coefficient is 0.74 (*p* < 0.001).

To explore this finding further, we also analyze the correlation between the topic similarity and the network density (the number of connections divided by the number of possible connections) [48]. The results reveal that there is a significant positive correlation (*r* = 0.79, *p* < 0.001) between the average topic similarity and the network density, and there is a weak negative correlation *(r* =  − 0.36, *p* < 0.001) between the topic similarity and the community size. Therefore, the more frequent the interaction between users, the greater the density and the efficiency of the users’ community, and the greater the similarity among the topics discussed.

Comparing the average topic similarity of the largest connected component in the community to the average topic similarity of all users in this community, we find that the theme similarity of the connected component is much greater than the theme similarity of the community (Fig. 4b). That is, after excluding the non-connected nodes, the theme similarity within a community increases. This is because there is a greater difference between the topics discussed by users who do not interact with each other.

### Community mining based on network topology

Based on network topology, we find a total of 1948 communities, of which 1605 are meaningful (excluding communities with only one node or without links). It can be observed that the degrees of connectivity of these communities are very high, and the proportion of fully connected (weakly) communities is 99.88%, i.e., 1603 out of the 1605 communities are themselves formed by nodes that are all weakly connected. Calculating the network efficiency and the average topic similarity of each topology-based community, we find a significant positive correlation (*r* = 0.73, *p* < 0.001, Fig. 4c), indicating that the greater the network efficiency, the higher the topic similarity within the community. This is in line with the finding concerning text-based communities above, validating the positive correlation between network efficiency and text theme similarity from a network perspective.

### Community-based text mining

**Fig. 5:**
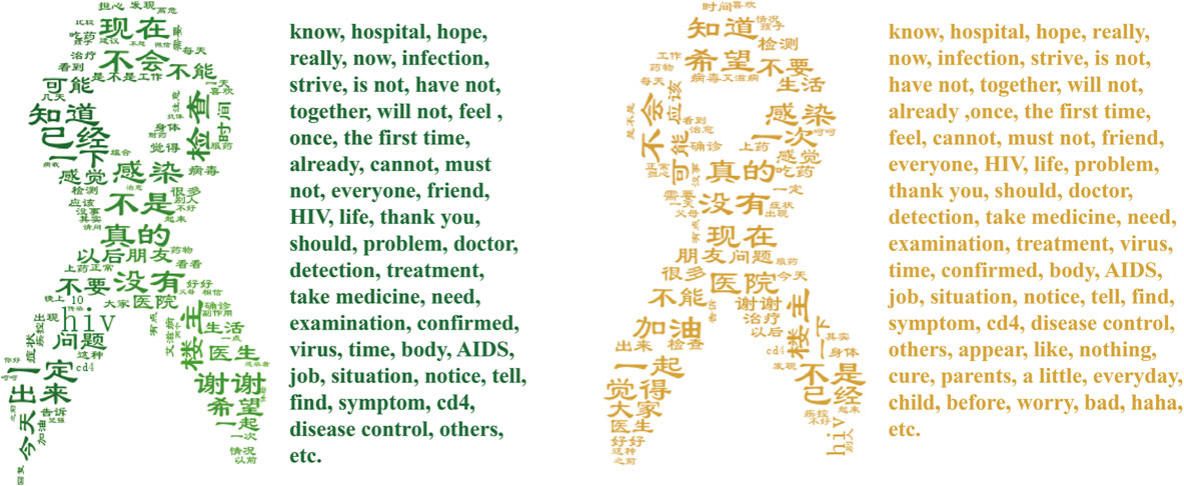
Common popular keywords appeared in more than 80% (left) and 50% (right) of HIV communities (see Additional file 1: Table S4 for details)

After extracting keywords for each community, we find that there is a significant overlap of popular keywords between different communities. The keywords appearing in most communities are presented in Fig. 5. We can see that words related to HIV/AIDS counseling and diagnosis, e.g., hope, infection, feel, may, know, appear very frequently. Most of the self-tagged HIV/AIDS patients are willing to share their physical states, as well as their own diagnosis or counseling on the online social network.

In addition, among these popular keywords, the negative words, e.g., do not, not, cannot, is not, appear frequently, indicating that there is a negative emotional tendency among the users in HIV/AIDS communities online. Moreover, most negative words are related to anxiety and fear of AIDS, e.g., “These symptoms make me worry, but I do not dare take a test.” It is worth noting that the phrase the first time occurs with high frequency, such as “The first time I checked HIV was in Shanghai Xinhua Hospital,” “For the first time I kissed a man, and then we got a room in the bathhouse [where people can sometimes call for sexual services in China],” “I drank last night and had WTGJ [the short form of the Chinese spelling for “anal sex without a condom,” i.e., high-risk behavior] for the first time.” This indicates that many people who suspect initial infection with HIV or have first contact with high-risk behavior tend to seek help and advice on a social networking platform in the beginning, rather than immediately going to a hospital for blood tests.

**Fig. 6:**
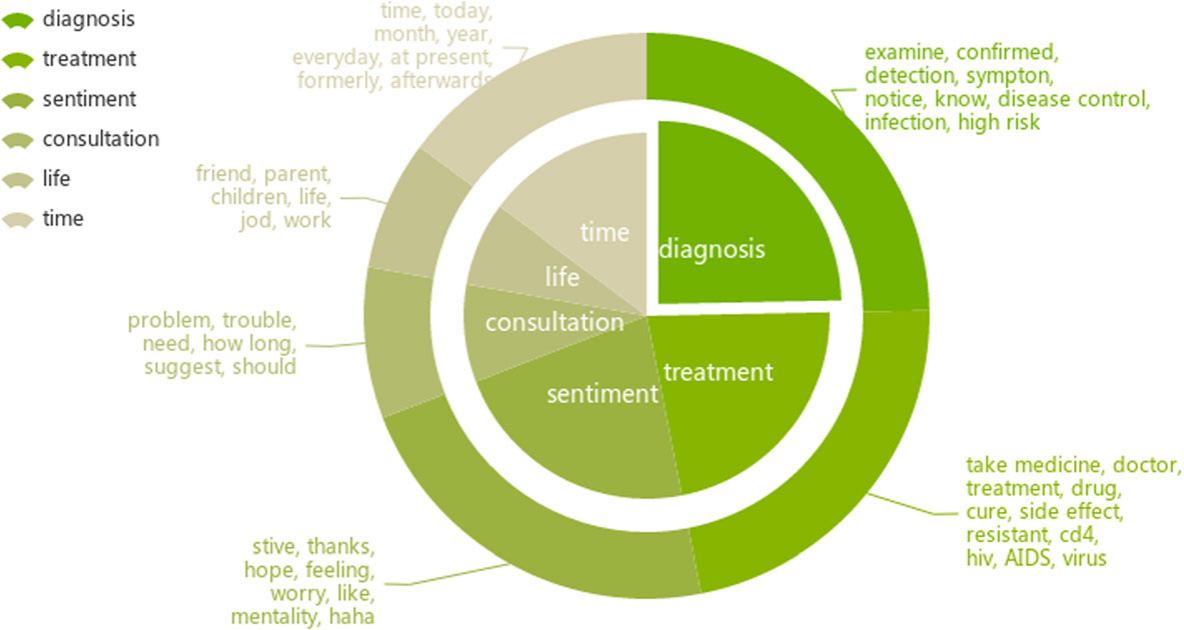
Distribution of topics in the HIV communities. Inner pie represents topics, and the outer ring represents popular keywords in each topic

We find a positive correlation between the topic similarity and the degree of interaction among community members. The closer the community members, the more similar the topics discussed in the community. Analysis of the topics discussed in these communities can reveal the needs and interests of HIV population. We analyze the top ten communities with maximum network efficiency and topic similarity values, and find that topics concerning HIV/AIDS diagnosis and treatment comprise a high proportion of the main topics of the HIV population, as shown in Fig. 6. In addition, it is worth noting that people tend to relieve their emotions in online communities, expressing their anxiety, horror, compunction, gratitude, or other feelings.

### Community-based sentiment analysis

**Fig. 7:**
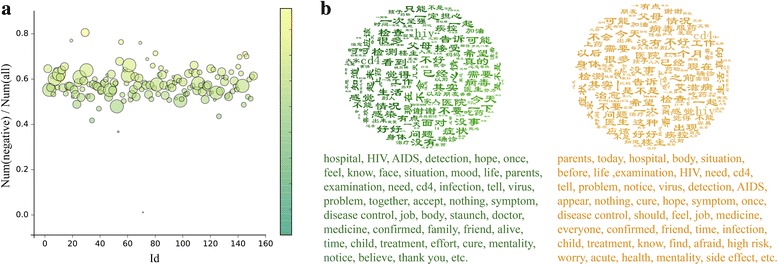
Figures in sentiment analysis. **a** The proportion of negative users in each community. The horizontal axis represents the community’s ID, the vertical axis represents the proportion of negative users in each community. Scatter size is proportional to the size of community and color corresponds to the ratio of negative users (green: low; yellow: high). **b** Popular keywords in different communities. Left: positive communities; right: negative communities (see Additional file 1: Table S5 for details)

Because sentiment analysis of communities based on network topology is very sensitive to the size of the community, we implement the analysis for each user community based on the results of text clustering. In Fig. 7a, we can see that in most communities the proportion of users with negative emotions is greater than 50%, indicating that most members’ emotions in these communities are negative. Moreover, the proportion of negative users in each community is around 60% and has a weak positive correlation with community size (*r* = 0.25, *p* = 0.002).

We select communities in which emotional tendencies are extreme, i.e., there are many more positive users in the community than negative users (hereinafter extreme positive community), or vice versa (hereinafter extreme negative community), to provide a comprehensive analysis of emotions. Specifically, we choose the top five communities in which positive users or negative users respectively account for the largest proportion, and extract the popular keywords posted in different communities according to the TF − IDF values. The results are shown in Fig. 7b, which shows that posts in extreme negative communities exhibit greater similarity, with a percentage of different popular keywords of only 35.75%; that is, 64.25% of the keywords discussed in all these communities overlap. In addition, we find that most of these keywords are about HIV/AIDS testing and treatment, physical condition, and family. In contrast, the percentage of different popular keywords is as high as 56% in extreme positive communities, and most keywords are about HIV/AIDS symptoms, counseling, testing, treatment, sentiment, and family. Comparing popular keywords between the extreme positive and extreme negative communities, we can see that in the extreme negative communities, more words are related to horror, anxiety, repentance, and other negative emotions, e.g., acute, high risk, side effects. However, in the extreme positive communities, users tend to express confidence, inspiration, gratitude, hope, and other positive emotions, and most popular keywords are about the HIV/AIDS diagnosis and active treatment.

## Discussion

### Summary of findings

In this paper, we analyze the mentality, behavior, and needs of the HIV population based on online communities formed by similar text content or by social interactions to understand the current living conditions and emotional status of the HIV/AIDS-related population online. Based on community data mining, we have found that there is a positive correlation between the average topic similarity of the HIV community and the degree of internal interactions; that is, users discussing similar topics are more likely to interact, and vice versa. In HIV communities, the topics of the online HIV groups are primarily related to HIV/AIDS diagnosis and treatment, and there is a domination of negative emotions in this community.

### Discussion of the main results

While it is a longstanding hypothesis that there is a correlation between similarity and friendship in human social activities [49–51], we demonstrate with real data that this is the case for online hidden populations: The degree of interaction and the topic similarity among users is positively correlated in HIV-related online communities. Moreover, this finding may provide insights for general social network studies, for which there may also be a relationship between interaction content and network topological structure.

This study reveals that most topics of concern to the online HIV community are related to HIV/AIDS testing, treatment, and HIV-related consultation, consistent with existing studies in which social support for the HIV population has been studied through text-only analysis [52]. And we also find that many users who suspect initial infection with HIV or have first contact with high-risk behavior tend to seek help and advice on social networking platform as their first choice. Because of the traditional conservative culture in China, people who are infected with HIV bear considerable social pressure and discrimination. In China, it is difficult to investigate the HIV population, and to understand their needs accurately through traditional survey methods. However, we have found that the main topics of the HIV group online are related to HIV/AIDS diagnosis and treatment, indicating that the HIV population tends to acquire HIV knowledge and seek help through online channels. These all supports the notion that we can provide more effective and timely help for the HIV population through text mining of data they post online, and it is important to improve support from online services for HIV/AIDS consultation and diagnosis to avoid privacy concerns and social discrimination.

Through sentiment analysis, we can see that negative emotions dominate in HIV communities, and these emotions are mostly related to the anxiety of initially infected patients, who tend to seek help and advice on social networking platforms as their first choice. To foster better social management, relevant agencies should pay more attention to the extreme negative communities. It is important to put these potentially HIV-infected groups under constant surveillance and to analyze their emotions continuously so that we can understand their needs, and provide relevant guidance and interventions promptly.

With the rapid expansion of Internet use in China, a large number of people who are interested in HIV-related topics are involved actively online nowadays. We have shown that there is great potential in extracting behavioral characteristics of such populations by analyzing the content and interaction networks generated online.

### Strengths and limitations

By analyzing the text content and social network of the HIV group from the largest Chinese online community, we have demonstrated the usefulness of online data mining for systematic investigation of the characteristics of hidden populations. There are several advantages with this methodology. First, the number of users in online communities is fairly large in comparison to the sample sizes achieved through traditional survey methods for hidden populations, such as people infected with HIV. Second, the richness of the data provided by online communities enables researchers to extract multidimensional characteristics of the target population, including features that are traditionally very hard to infer, e.g., social networks, emotional needs, etc. Third, the anonymity of online communities mitigates the privacy concern, and users can express their views at liberty, which ensures the accuracy of studies on hidden populations.

However, it is worth noting that it remains to be validated that whether the findings concerning users in online communities can be extrapolated to the target population in real life. The representativeness of the online populations in topic-specific communities, differences in population characteristics across social networking platforms, and the design and implementation of public health intervention strategies are yet to be studied in the future.

## Conclusion

By analyzing the text content and social network of the HIV group from the largest Chinese online community, we have demonstrated the usefulness of online data mining for systematic investigation of the characteristics of hidden populations, including temporal patterns of online activity, social network, community structure and its connection to social distance and similarity of content, emotional tendency, etc. The methodology is of particular importance to China, which is experiencing a heavy burden of HIV infection, with surprisingly high number of new infections among certain populations such as MSM [53]. The rapid expansion of Internet use and increasing online engagement thereby offer new opportunities for the study of hidden population with unprecedented sample sizes and richness of information. Our study also suggests that public health agencies should promote education online to reduce high risk behaviors and expand channels for HIV/AIDS counseling and testing such that those who are suspecting of initial infection could seek for advice. In addition, psychological counseling and guidance for HIV/AIDS patients are also in need, as newly infected patients are greatly worried about their condition and are psychologically fragile [54].

## Additional file

Additional file 1: Supplementary Information. (DOCX 55 kb)

## Abbreviations

AIDS: Acquired immunodeficiency syndrome
HDP: Hierarchical Dirichlet Process
HIV: Human immunodeficiency virus
LDA: Latent Dirichlet Allocation
MSM: Men who have sex with men
