# Supplementary material for: Analyzing hidden populations online: topic, emotion, and social network of HIV-related users in the largest Chinese online community
Source: BMC Med Inform Decis Mak. 2018 Jan 5;18:2. doi: 10.1186/s12911-017-0579-1 (PMC5755307; doi:10.1186/s12911-017-0579-1)
Supplement: Additional file 1: — Supplementary Information. (DOCX 55 kb) [file 12911_2017_579_MOESM1_ESM.docx]

**Additional file**

Table S1 Comparison of the sentiment analysis results with human judgments

| **Corpus** | **Translation** | **Emotional Tendency** | | |
| --- | --- | --- | --- | --- |
|  |  | **Dictionary** | **Human A** | **Human B** |
| 无聊到爆今天睡了一天。吃药第12天明天要去疾控了 | It is so boring that I slept the whole day. I have taken medicine for 12 days and will go to the disease control center tomorrow. | N | N | N |
| 阳就阳了呗。这么多阳的也没有死呢。阳了就阳光点 | It is not a big deal of being positive. There are so many people living with it. If you are one of them, keep active in life. | N | P | P |
| 讨厌。人家都哭了 | Annoying. I am crying. | N | N | N |
| 在这鸟地方。没人问津，感染者就我一个上贴吧。想找人聊聊都没有。真是无聊翻了 | This place is awful. No one is interested in visiting here and I’m the only one posting, without anyone to talk to. Really boring. | N | P | N |
| 只有妈妈在身边。她难受我也难受。我难受她也难受 | My mother is the only one who accompanies me. We are both sad whenever who is suffering. | N | N | N |
| 那你去死吧。祝你死的好看点 | Go to hell! I wish you die with a good looking. | N | N | N |
| 这不是没钱么。在家闲着没事干 | I do not have money and have nothing to do at home. | N | N | N |
| 那多残忍。多暴力 | That is cruel and violent. | N | N | N |
| 哈哈哈。那你加油吧。祝你早日康复 | Hahaha. Come on! I wish you recover soon. | P | P | P |
| 妈妈每天都难过。我都不敢看 | My Mom is sad every day. I do not dare to even look at her. | N | N | N |
| 是病总会有可以被治愈的那一天,一定要相信未来 | There will be a day for any disease to be cured, please believe in the future. | P | P | P |
| 好好照顾自己亲 | Take good care of yourself, dear. | P | P | P |
| 希望每一个朋友都能向他们一样幸福 | I hope every friend can be as happy as them. | P | P | P |
| 坚强,为了孩子,如果你不在了孩子会更苦,加油 | Be strong for your children! They will be more miserable if you die, cheer up! | P | P | P |
| 阿姨加油、相信他会好起来的。 | Cheer up Aunt, I believe that he will get well. | P | P | P |
| 死基佬，这是报应 | Damn gay, this is retribution. | N | N | N |
| 身为HIV感染者的我、都超级讨厌同性恋 | I hate gay, even I’m also infected [with HIV]. | N | N | N |
| 自恋什么啊、既不漂亮、又跟我一样是感染者 | There is nothing to be narcissistic. You are not pretty, and are also infected as I am. | P | N | N |
| 得了这个病还到处找BF、不知道会交叉感染、好好爱家人不是很好、为什么还要去害人 | Infected with this disease yet not knowing of cross-infection, finding boyfriends everywhere, why don’t you love family instead of hurting people? | P | N | N |
| 可能是心理因素,我还是蛮担心的 | Maybe it’s psychological, I am still quite worried. | N | N | N |
| 孩子，你才18岁，要好好活着。不久的将来会有药物能治疗的，不抛弃不放弃 | Child, you are only 18 years old, stay alive. There will be drugs to treat the disease in the near future, do not give up. | P | P | P |
| 石榴兄弟，我感觉你一定会活到有特效药的哪一天，我最近看新闻特别多hiv这方面的突破，你这么乐观，可以多帮助一下我们这些恐友，我不大会说话，怕说的话会伤害到你 | Pomegranate brother, I can feel that you will surely live to the day of cure. Recently I read lots of news about the breakthrough in HIV treatment. Your positive attitude can help us. I am not good at expression but I hope I’m not hurting you. | P | P | P |
| 他心态不好 | He has a bad mentality. | N | N | N |
| 谢谢楼上的朋友！祝你明天鸿运当头！ | Thank you, friend! Good luck tomorrow! | P | P | P |
| 试着去喜欢这样的感受，加油 | Try to enjoy this feeling, cheer up! | P | P | P |
| 一切顺其自然，许愿大家都安好 | Everything goes its way, I wish everyone is well. | P | P | P |
| 中药长期吃会有严重的副作用 | Long-term Chinese medicine taken has serious side effect. | N | N | N |
| 我本来不想说什么的，因为理解你的心情，大家都恐我也恐的要死！现在也特害怕，但是你每天总发这样的帖子，还没有发生，你非要制造这种情绪就不应该了，兄弟！ | I did not want to say anything, as I understand your feelings. Everyone has a fear of HIV infection, and so do I! But you should not always post such messages and make this unwelcomed atmosphere out of nothing, brother! | N | N | N |
| 好样的！ | Well done! | P | P | P |
| 日有所思夜有所梦，正常的，心情太紧张，越希望的事情越会在梦境里出现，都是自我暗示，你现在放宽心，好好吃药，乖乖睡觉，都会好的 | Dreaming during the day, there is a dream at night. It is normal, as all are self-suggestion such that when you are too nervous, the more you want, the more you will dream. Now you should relax, take medicine, and sleep well. Everything will be fine. | P | P | P |
| 不要理质疑你的人，你的文章写的很棒，记得一定要注意自己的身体，找个没夜班的工作，营养一定要加强，缺钙了买瓶钙尔奇碳酸钙D，你一定要加油，不能自暴自弃，还有很多人关心你的！ | Ignore those who question you. Your article is great. Remember to take care of yourself, find a job without night work, and strengthen nutrition. Come on! Do not give up, there are a lot of people who care about you! | P | P | P |
| 不是怕传染，而是如果这事传开了，你女儿以后会受到各种歧视，对她成长很不利。你想想上学时候他们叫你女儿艾滋女，她是什么心情 | Being infected is not the thing that you should be afraid of. If the fact [of being infected] is known to others, your daughter will be subjected to various discrimination, which is harmful for her growth. Try to think what she feels when people call her AIDS-daughter. | N | N | N |
| 你永远叫不醒一个装睡的人。何必理他们呢 | You can never wake up someone who pretends to sleep. Why bother them? | N | N | N |
| 这两天虽然我身体没有不适但心灵却饱受煎熬我是罪人，自从那天以后到现在我天天去天主教堂忏悔，我才19岁，而且是大学生，我不能就这样毁了，主啊，我已经知道错了，上帝会原谅年轻人犯这种罪吗？ | Although I have no discomfort for these two days, my heart is suffering. I am a sinner. Since then I went to the Catholic Church to repent every day. I am a 19 years old college student. I cannot ruin my life like this. God, I already know my fault. Will God forgive young people who made such a mistake? | N | N | N |
| 心烦睡不着，后天复查加全身检查 | [I am] Upset and cannot fall in sleep. I will re-visit the hospital with a body check the day after tomorrow. | N | N | N |
| 也许是真的累了，每天就像行尸走肉一样，失去了动力，真的不知道还能坚持到什么时候，希望可以遇到人生的另一半，以后会好好对她的，本人25岁，在合肥，希望可以遇到安徽及周边的女生！ | I might be really tired, like a walking dead without any motivation every day. I don’t know how much longer I can carry on. I hope I can meet the partner of my life, I will take good care of her. I’m 25 years old, in Hefei, hope to meet girls in Anhui or the surrounding! | P | P | P |
| 正能量个屁！无知的人类，这病为什么会得？是天作孽还是自作孽？人们不乱搞性开放，不搞婚前性，这病会这么嚣张吗？不觉得是一种惩罚吗？我敢保证如果人类继续这样下去，即使这个疫苗成功了，病毒也会突变，到时想真正治愈那是不可能的！此帖为证，到时可以来见证我说的对不对！ | Positive energy is bullshit! Ignorant human. Why did you get this disease? Should you blame God or blame yourself? How could the disease be so prevalent without people engaging in sexual openness and premarital sex? Don’t you think it is a punishment? I can assure that if human continue to behave like this, even if the vaccine is successfully developed, the virus will mutate, and it will be impossible to cure the disease! This post testifies my words! | N | N | N |
| 挺伤感的，我哭了，感觉很不是滋味！今天走到这个吧，感觉很心痛，我看到蛮多帖子。16岁，17岁，18岁那是那么的年轻啊！！ | I cried and felt bad! I ran into this bar today, felt very sad after reading a lot of posts. [Many of them are] 16, 17, 18 years old, so young!! | N | N | N |
| 明明知道错了难道上帝不能给一次机会吗？？ | Obviously, I already know my fault. Cannot God give me a chance?? | P | N | N |
| 与其颓废，消极的生活，还不如珍惜，感悟，拼搏的生活 | Live with cherishing and hardworking rather than with a passive attitude. | N | P | P |
| 加油，一起面对！ | Come on, let’s face the challenge together! | P | P | P |
| 我喜欢和别人聊天，我觉得和别人交流一下，心里会舒服很多，也很感谢那些帮助过我的朋友，也希望我能帮助到别人。有想聊一聊的可以加我QQ286041726 | I like to chat with people. After communicating with others, I feel much more comfortable. I am grateful to those who helped me and I also hope I can help others. You may add my QQ286041726 [QQ is a popular instant messaging software in China] if you want to talk. | P | P | P |
| 瞬间笑了，你这个缺心眼的家伙 | In a flash, I laughed. You're a careless guy. | N | P | P |
| 我也曾经跟父母争吵想过自杀。不过我还是心痛我妈，她不能那么惨。我对不起我爸妈 | I was thinking of suicide when I quarreled with my parents. But I felt bad for my mother, she should not be so miserable. I'm sorry to my parents. | N | N | N |
| 你见到谁怨天尤人了吗？这个吧还是有很大的正能量的，忘记这个病自己也能很阳光的活下去，其实本来没什么，是你自己的心态不好 | Who do you see is complaining? This bar [the HIV bar] is filled with positive attitudes. Forget the disease, you can still live actively. In fact, it’s nothing but your bad mentality [in your posts]. | N | P | P |
| 做点有意义的事情，帮父母干点什么，或者是培养自己点兴趣爱好，或者去哪玩玩调整一下自己的心态，这吧里很多病友很多比你还要惨不知道多少倍多 | Do something meaningful, help your parents, develop some hobbies, or travel somewhere to adjust your spirit. There are a lot of patients who have much worse situation than you. | P | P | P |
| 心疼，一路走好。 | Distressed, wish you rest in peace. | N | P | N |
| 不要轻易放弃，人生很美好，坚持服药，一定可以坚持到能被治愈的那天的 | Do not give up easily, life is wonderful. You must be able to stick to the day of being cured if you insist on taking medicine. | P | P | P |
| 今年在家被逼婚逼的急了就坦白了，老爸感觉一下老了好多。心里感觉很复杂 | I confessed [the HIV infection] to my parents this year when I was pushed to get married. My dad looked much older at a moment. My feeling is very complicated. | N | N | N |
| 我的父母也是，每天装做若无其事，家里的用具都是分开使用，有的时候很想自杀，我的CD4才51，现在还没服药，可我觉得就这样走很是自私，生虽然不能给他们带来快乐，但死只会给他们带来无尽的悲伤，有的时候我也不知道该怎么办 | My parents are the same, pretend that nothing had happened. Household appliances are used separately at home. Sometimes I want to commit suicide, my CD4 is only 51. I have not taken medicine. But I feel that it’s too selfish if I kill myself. Although I cannot bring them happiness when I am alive, but my death would bring them endless sorrow. Sometimes I do not know what to do, neither [echoing what the previous post says]. | N | N | N |
| 我只希望父母发发牢骚就过去了，过去了就不再想了，看着他们都消瘦了，我的心也真的很难受，我该怎么办？怎么才能让他们更加顺心 | I just hope my parents complain and forget, no longer think over it. Looking at them becoming thinner and thinner, I’m very upset. What should I do? How to make them feel better? | N | N | N |
| 祝楼主新婚快乐，希望能给你冲冲喜，将这病冲走 | I wish you a happy wedding and hope the marriage brings you good luck and wash the disease away. | P | P | P |
| 一定要冷静对待，不要被气昏了头脑 | Stay calm, do not get angry or mad. | N | P | P |
| 我知道，可你也别糟践自己的身体，酒能不喝就不喝，心情不好的时候可以和我们诉说，别再喝闷酒了 | I know, but you shouldn’t hurt yourself. Drink as less as possible. Chat with us if you are in a bad mood. Do not drink alone anymore. | N | P | P |
| 我的父母很注重自己的脸面，我是他们的耻辱，让他们在人前抬不起头，有的时候不是我想颓废，而是这个社会将我逼成这样的 | My parents care much about their reputation. I am the one who made them ashamed. Sometimes it’s not that I want to be decadent, it’s the society which forced me to be like that. | N | N | N |
| 我今天真的很想哭，看着跟我有同样病的病友们在传院领药，心里有种说不出的滋味，他们有的人真个身体都发黑，我真的害怕我以后也会变成那个样子 | I really want to cry today. When I see patients who have the same disease receiving medicine in the hospital, I feel very complicated. Some of them are black all over their body. I am really scared that I will be like that someday. | N | N | N |
| 不是我看的开，我要是再看不开点儿，我非得抑郁死，但愿能有一日攻克这个疾病 | It is not because I am a positive person. If I am not, I might have been depressed to death. I hope the disease can be cured in the future. | N | N | P |
| 说真的那些嫖娼，约炮，基佬得艾滋病的都不值得同情，说句不好听的，自己找死，死了活该了 | Those who are infected with HIV by prostitution, one-night stand, or homosexuality are not worth of sympathy. To be honest, this is what they deserve. | N | N | N |
| 楼楼长的好漂亮 | The poster is very beautiful. | P | P | P |
| 和我这是感染吗？我好怕，真的好怕！现在晚上都睡不着了！ | Am I infected? I'm so scared, really scary! Now I cannot fall in sleep at night! | N | N | N |
| 知道阳了后，和男友分手了，怕伤害他。我的心好疼，每天就要喝醉才能入睡 | I broke up with my boyfriend after knowing of being positive. I am afraid of hurting him. My heart hurts and I get drunk every day before sleep. | N | N | N |
| 楼主压根是个疯子，无知，可笑 | The poster is crazy, ignorant, and ridiculous. | N | N | N |
| 那一刻 你怕吗 我现在就怕每天看到父母劳累的样子 | Are you scared at that moment? I am afraid of seeing my parents’ tired looking now. | N | N | N |
| 他哭闹的我心疼，没分手成功 | I was distressed by his crying, did not break up successfully. | N | N | N |
| 楼主，看完你的故事了，很受感动。希望你坚强，一切都会有好转，我相信这个病无法完全治愈，但是只要坚持治疗，再加上现在医学这么发达，一定有更多更好的药出来，不要低头，加油 | Poster, after reading your story, I am very touched. I hope you be strong, and everything will be better. Although this disease cannot be completely cured, there must be more and better medicines being developed, as long as we stick to treatment. Do not be depressed, cheer up! | P | P | P |
| 你这种尖酸刻薄的东西枉为人，我诅咒你全家不得好死 | You are so mean, I curse your whole family to die. | N | N | N |
| 感觉整个人就像行走的僵尸，身心都被掏空了！但无论怎样，生活还要继续！一起加油 | I feel like a walking zombie. Both my body and soul have been hollowed out! But no matter what, life should continue! Let’s work hard together! | P | P | P |
| 争吵中他推了我，然后我又推了他，然后他又踹了我，然后我又踹他，但没有踹到，然后我又一拳打在他的脸上，然后我俩摔在一起，在地上混战，最后我拿杯子扔他脸上了，鼻子出了好多血，以为骨折了 | In the quarrel he pushed me, and I pushed him back, then he kicked me, and I kicked back, but I did not reach him. I punched him in the face, then we both fell and flighted on the floor. At last I threw a cup on his face, a lot of blood was flowing from his nose, and I thought he had a fracture. | N | N | N |
| 告诉你一个特别可怕的副作用。。。长期服用后会从聪明伶俐，变成脑残，比如像我。。。。 | I’m telling you a terrible side effect… After long-term medication, intelligent people will become stupid, such as me. . . | N | N | N |
| 也希望楼主能够做更多有意义的事情,帮助他人~ | I also hope the poster can do more meaningful things to help others ~ | P | P | P |
| 还有一点奉劝，远离高危，切记！！！ | Here is another advice: stay away from high-risk behavior, remember! ! ! | N | P | N |
| 都一样！向自己的目标前进时，总会碰到各种各样的障碍，阻挡前行，我们要想办法去化解它，只要坚持就能做到！ | It's the same! We must find solutions to remove the obstacles which are in the way to our goal. If we persist, we can make it! | P | P | P |
| 我持续腹泻，头晕无力肌肉疼，体重下降，我也是肠子都悔青了。以后绝对不再高危了 | With continuing diarrhea, dizziness, muscle weakness and weight loss, I regret so much and will never try high-risk behaviors again. | N | N | N |
| 兄弟，我他妈的家里有那么多牵挂都不死，你死毛啊，你父母不在生活没多少压力，不用愧对父母，好好的活啊 | Man, I have so many families and I’m still fucking alive, why do you choose to die? Since your parents have passed away, you don’t have much pressure in life, live well! | N | P | P |
| 我已经愤怒了！彻彻底底的愤怒了！疫苗在哪里？干预政策又在哪里？？如果国家早点重视了，身边的朋友和素不相干的同胞还会有不断的被感染的吗？ | I'm angry! Completely angry! Where is the vaccine? Where is the intervention policy?? If the government could pay earlier attention to this issue, will my friends and compatriots continue to be infected? | N | N | N |
| 我好怕 | I am scared. | N | N | N |
| 你的家里人只是嘴上说抛弃，心里是绝对不会抛弃的，加油 | Your family just say that they abandon you, but they never mean to it! Cheer up! | N | P | P |
| 非常幸运，我总算真正的获得了重生！ | Very lucky, I finally got a real rebirth! | P | P | P |
| 李老师祝你新的一年快快乐乐，健健康康，您真是好人～ | Teacher Li, I wish you a happy and healthy new year, you are really nice ~ | P | P | P |
| 加油！！！不要放弃自己，人都是求生的 | Come on!!! Do not give up. We all want to be alive. | N | P | P |
| 只要好好活着就有治愈的希望，楼主加油啊 | As long as being alive, there is hope of cure. Cheer up. | P | P | P |
| 我会加油的的...会好好过下去 | I will cheer up... and live well. | P | P | P |
| 重感情要找对人，，我也刚刚分手，，觉得自己弄得自己很痛苦 | If you value your love, find the right lover,, I just broke up,, and feel that I made myself so painful. | N | N | N |
| 你们要加油哦！！祝你们永远幸福快乐！ | You must cheer up!! I wish you happy forever! | P | P | P |
| 他们都会遭到报应的，一群没良心的东西，赚这种黑心钱，迟早只能买棺材！ | This is a group of people with no conscience, they will get their own retribution. The black-heart money will only be used to buy them coffins! | N | N | N |
| 哎，后悔没有用呀，现实是残酷的 | Ah, regret is useless, the reality is cruel. | N | N | N |
| 恩恩，谢谢你，大家都保重哟 | Well, thank you, everyone takes care of yourself! | P | P | P |
| 为什么不爱惜自己呢，患上这样的病真的很让人难过 | Why don’t you care about yourself, suffering from such a disease is really sad. | N | N | N |
| 我好难受，想起妈妈的笑容我就难受 | I feel so sad each time when I think of my mother's smile. | N | N | N |
| 我现在盗汗好严重，低热，我会不会很快就死掉，等不到半个月后用药了，我好害怕 | I have serious night sweats and fever now. Will I die soon? I cannot wait for half a month before taking medicine. I'm so scared. | N | N | N |
| 心里害怕所以一直没去医院问结果 | I’m too scared to ask the result in the hospital. | N | N | N |
| 所以,罪魁祸首,还不就是你这种人渣! | So the culprit are scumbags like you! | N | N | N |
| 我也要急疯了 我还是学生 没钱自费 听说上药后还会有副作用 还得自费 真是恨透了自己 | I am crazily anxious. I am a student who do not have much money. I heard that there are side effects after taking the drugs, and some drugs are at own expense. [I] Really hate myself. | N | N | N |
| 很难受，等待最后几天的结果，和大家一起战斗 | I’m very upset when waiting for the results for the last few days. Be strong together! | N | N | N |
| 你这帖子太暖心了，以后我难过了就来看看你的贴，让我有活下去的勇气。你很坚强，我感觉我一个男孩子都不如你 | Your post is heart-warming. I will come back to read your posts when I feel upset. It gives me courage to live. Even you are a girl, I feel that I’m not as strong as you. | P | P | P |
| 坚强起来 真的 要相信自己 | Be strong, seriously, believe in yourself. | P | P | P |
| 是经常悔恨自责，是不是快抑郁了。 | I often feel regret and condemn myself, am I almost in depression? | N | N | N |
| 虽然这个是不孝顺。可是你爸是真的爱你吗？本来就命不久矣，很多人都想要自杀，他还火上浇油，不怕你做出傻事来吗？我看你爸也是没良心，赌徒一般都没什么良心的。 | Although this is unfilial. But does your dad really love you? With this disease we don’t have much time to live, and many people want to commit suicide. But your dad still pushes you, is he not afraid of you doing stupid things? I don’t think he has conscience. Gamblers usually have no conscience. | P | P | N |
| 希望上天保佑他。没有父母的疼爱，本来就很可怜。希望这次他度过难关。加油！ | God bless him! Without parents’ love, he is very pitiful already. I hope he will overcome these difficulties this time. Come on! | P | P | P |
| 我也想喝中药，西药让我一直犯恶心，好讨厌 | I want to take Chinese medicine as well. Western medicine always makes me nauseated, so annoying. | N | N | N |

Notes: P represents positive emotions, and N represents negative emotions.

Table S2 Precision and recall of the sentiment analysis results, human A as the gold-standard

|  | **Positive** | **Negative** |
| --- | --- | --- |
| Positive | 35 | 12 |
| Negative | 3 | 50 |
| Precision | 85% | |
| Recall | 92.1% | |

Table S3 Precision and recall of the sentiment analysis results, human B as the gold-standard

|  | **Positive** | **Negative** |
| --- | --- | --- |
| Positive | 34 | 10 |
| Negative | 4 | 52 |
| Precision | 86% |  |
| Recall | 89.5% |  |

Table S4 Common popular keywords appeared in most of HIV communities

| **Common keywords in 80% clusters** | | | **Additional keywords in 50% clusters** | | |
| --- | --- | --- | --- | --- | --- |
| Keywords | Translation | Frequency | Keywords | Translation | Frequency |
| 知道 | know | 1.00 | 别人 | others | 0.83 |
| 医院 | hospital | 1.00 | 出现 | appear | 0.79 |
| 希望 | hope | 1.00 | 喜欢 | like | 0.77 |
| 真的 | really | 1.00 | 没事 | nothing | 0.76 |
| 没有 | have not | 0.99 | 父母 | parents | 0.75 |
| 现在 | now | 0.99 | 有点 | a little | 0.70 |
| 感染 | infection | 0.99 | 每天 | everyday | 0.70 |
| 加油 | come on | 0.99 | 正常 | normal | 0.69 |
| 不是 | is not | 0.99 | 治愈 | cure | 0.65 |
| 一起 | together | 0.99 | 孩子 | child | 0.60 |
| 不会 | will not | 0.99 |  |  |  |
| 觉得 | think | 0.99 |  |  |  |
| 一次 | once | 0.99 |  |  |  |
| 已经 | already | 0.99 |  |  |  |
| 不能 | can not | 0.99 |  |  |  |
| 不要 | do not | 0.99 |  |  |  |
| 大家 | everyone | 0.99 |  |  |  |
| 朋友 | friend | 0.99 |  |  |  |
| HIV | HIV | 0.99 |  |  |  |
| 生活 | life | 0.99 |  |  |  |
| 谢谢 | thanks | 0.99 |  |  |  |
| 感觉 | feel | 0.99 |  |  |  |
| 应该 | should | 0.99 |  |  |  |
| 问题 | problem | 0.99 |  |  |  |
| 医生 | doctor | 0.98 |  |  |  |
| 检测 | detection | 0.98 |  |  |  |
| 治疗 | treatment | 0.98 |  |  |  |
| 吃药 | take medicine | 0.97 |  |  |  |
| 检查 | examination | 0.97 |  |  |  |
| 以后 | after | 0.97 |  |  |  |
| 确诊 | confirmed | 0.97 |  |  |  |
| 需要 | need | 0.96 |  |  |  |
| 一定 | certain | 0.95 |  |  |  |
| 病毒 | virus | 0.95 |  |  |  |
| 时间 | time | 0.95 |  |  |  |
| 今天 | today | 0.95 |  |  |  |
| 身体 | body | 0.95 |  |  |  |
| 艾滋病 | AIDS | 0.93 |  |  |  |
| 工作 | job | 0.91 |  |  |  |
| 情况 | situation | 0.91 |  |  |  |
| 告诉 | tell | 0.91 |  |  |  |
| 发现 | find | 0.91 |  |  |  |
| 症状 | symptom | 0.90 |  |  |  |
| cd4 | cd4 | 0.90 |  |  |  |
| 药物 | medicine | 0.85 |  |  |  |
| 疾控 | disease control | 0.84 |  |  |  |
| 其实 | actually | 0.83 |  |  |  |

Table S5 Popular keywords in different sentiment communities

| **Popular keywords in negative communities** | | | **popular keywords in positive communities** | | |
| --- | --- | --- | --- | --- | --- |
| Keywords | Translation | Frequency | Keywords | Translation | Frequency |
| 父母 | parents | 1.00 | 医院 | hospital | 1.00 |
| 一起 | together | 1.00 | hiv | HIV | 1.00 |
| 今天 | today | 1.00 | 检测 | detection | 1.00 |
| 医院 | hospital | 1.00 | 希望 | hope | 1.00 |
| 身体 | body | 1.00 | 真的 | really | 1.00 |
| 情况 | situation | 1.00 | 一次 | once | 1.00 |
| 之前 | before | 1.00 | 觉得 | feel | 1.00 |
| 生活 | life | 1.00 | 知道 | know | 1.00 |
| 检查 | examination | 1.00 | 面对 | face | 0.75 |
| HIV | HIV | 1.00 | 情况 | situation | 0.75 |
| 需要 | need | 1.00 | 心情 | mood | 0.75 |
| cd4 | cd4 | 1.00 | 生活 | life | 0.75 |
| 告诉 | tell | 1.00 | 父母 | parents | 0.75 |
| 问题 | problem | 1.00 | 检查 | examination | 0.75 |
| 注意 | notice | 1.00 | 需要 | need | 0.75 |
| 病毒 | virus | 1.00 | cd4 | cd4 | 0.75 |
| 检测 | detection | 1.00 | 感染 | infection | 0.75 |
| 艾滋病 | AIDS | 1.00 | 告诉 | tell | 0.75 |
| 出现 | appear | 1.00 | 问题 | problem | 0.75 |
| 没事 | nothing | 1.00 | 一起 | together | 0.75 |
| 治愈 | cure | 1.00 | 病毒 | virus | 0.75 |
| 希望 | hope | 1.00 | 接受 | accept | 0.75 |
| 症状 | symptom | 1.00 | 没事 | nothing | 0.75 |
| 已经 | already | 1.00 | 症状 | symptom | 0.75 |
| 真的 | really | 1.00 | 谢谢 | thanks | 0.75 |
| 一次 | once | 1.00 | 疾控 | disease control | 0.75 |
| 疾控 | disease control | 1.00 | 工作 | job | 0.75 |
| 应该 | should | 1.00 | 担心 | worry | 0.75 |
| 感觉 | feel | 1.00 | 身体 | body | 0.75 |
| 工作 | job | 1.00 | 坚强 | strong | 0.75 |
| 药物 | medicine | 1.00 | 医生 | doctor | 0.75 |
| 大家 | everyone | 1.00 | 上药 | medication | 0.75 |
| 谢谢 | thanks | 1.00 | 确诊 | confirmed | 0.75 |
| 确诊 | confirmed | 1.00 | 家人 | family | 0.75 |
| 朋友 | friend | 1.00 | 朋友 | friend | 0.75 |
| 时间 | time | 1.00 | 活着 | alive | 0.75 |
| 感染 | infection | 1.00 | 时间 | time | 0.75 |
| 孩子 | child | 1.00 | 孩子 | child | 0.75 |
| 治疗 | treatment | 1.00 | 治疗 | treatment | 0.75 |
| 知道 | know | 1.00 | 努力 | effort | 0.50 |
| 发现 | find | 0.75 | 治愈 | cure | 0.50 |
| 害怕 | afraid | 0.75 | 心态 | mentality | 0.50 |
| 容易 | easy | 0.75 | 注意 | attention | 0.50 |
| 传染 | infection | 0.75 | 相信 | believe | 0.50 |
| 担心 | worry | 0.75 |  |  |  |
| 比较 | compare | 0.75 |  |  |  |
| 高危 | high risk | 0.75 |  |  |  |
| 急性 | acute | 0.50 |  |  |  |
| 健康 | health | 0.50 |  |  |  |
| 心理 | mentality | 0.50 |  |  |  |
| 副作用 | side effect | 0.50 |  |  |  |
